# Dynamics and Assembly Mechanisms of Bacterial Communities During Larval Development of *Macrobrachium rosenbergii*: A High-Frequency Sampling Study Based on 16S rRNA Absolute Quantification Sequencing

**DOI:** 10.3390/microorganisms13081881

**Published:** 2025-08-12

**Authors:** Zhibin Lu, Jingwen Hao, Jilun Meng, Cui Liu, Tiantian Ye, Junjun Yan, Guo Li, Yutong Zheng, Pao Xu, Zhimin Gu

**Affiliations:** 1 Xianghu Laboratory, Hangzhou 311231, China; luzhibin@xhlab.ac.cn (Z.L.);; 2 Wuxi Fisheries College, Nanjing Agricultural University, Wuxi 214081, China

**Keywords:** *Macrobrachium rosenbergii*, larviculture, absolute quantification sequencing technology, bacterial community, High-frequency sampling, abundance–occupancy model

## Abstract

This study aimed to elucidate stage-specific dynamics, assembly mechanisms, and functional roles of bacterial communities during *Macrobrachium rosenbergii* larval development through high-resolution microbiota profiling. A high-frequency sampling strategy (126 samples across 11 zoeal stages and 1 post-larval stage within 21 days) and 16S rRNA absolute quantification sequencing were employed. Bacterial succession, persistent taxa, and ecological processes were analyzed using abundance-occupancy modeling, neutral community modeling, and PICRUSt2-based functional prediction. Absolute bacterial abundance exhibited a triphasic abundance trajectory. Initial accumulation: Linear increase (Dph 1–5, peak Δlog10 = +1.7). Mid-stage expansion: Peak abundance (log10 = 7.5 copies/g, Dph 7–8). Late-stage remodeling: Secondary peak (log10 = 7.1 copies/g, Dph 19). Eighty dominant amplicon sequence variants (ASVs) (dominant taxa: *Herminiimonas*, *Maritalea*, and Enterobacteriaceae) comprised > 95% of the total abundance and coexisted via niche partitioning. Community construction was dominated by ecological drift/dispersal limitation (neutral model R^2^ = 0.16, *p* < 0.01). Metabolic pathways (e.g., nutrient metabolism) shifted with dietary transition. “Phylogenetic replacement” underpinned microbiota resilience against environmental perturbations. Optimizing aquaculture environments offers a viable antibiotic-free strategy for microbial management, advancing our understanding of host microbe interactions and ecological niche differentiation in aquatic animals.

## 1. Introduction

The giant freshwater prawn (GFP; *Macrobrachium rosenbergii*) holds a central position in global aquaculture, renowned for its high nutritional value and substantial economic contributions [1]. Its larval development follows a sequential process encompassing eleven zoeal stages (zoea I–zoea IX) and 1 postlarval stage [2]. Over the past decade, a growing body of research has highlighted the critical role of microbiota in determining the viability of aquatic animal larvae [3,4]. Multiple studies have demonstrated that microbiota established during early life stages not only shape later-life microbial communities but also exert long-term impacts on host fitness, including health, development, and disease susceptibility [5,6,7,8]. For instance, Bacillota can enhance nutrient absorption and inhibit pathogens through antimicrobial substances [7,9]; *Actinomycetota* is capable of producing enzymes that support the digestion of complex diets [10]; meanwhile, *Maritalea* exhibits the ability to degrade polysaccharides (e.g., chitin) and synthesize vitamins, which may contribute to nutrient supply during larval development [11]. However, GFP infected with *Enterobacter cloacae* demonstrated significantly retarded growth rates, accompanied by characteristic physiological manifestations associated with the pathogenic impact of this bacterium [12]. Typically, the larval microbiota exhibits lower stability and maturity compared to that of juveniles or adults [6]. In nursery water, low salinity and high ammonia nitrogen levels enhance the risk of r-selected bacteria, such as *E. cloacae* and *Vibrio parahaemolyticus*, which are recognized as primary drivers of elevated mortality in GFP larvae [13,14]. Previous studies have investigated the development of GFP larval microbiota using relative qualification of 16S amplicon sequences with a relatively low sampling frequency [9,10,15]. These studies revealed that the microbial community structure in water at 7, 14, and 21 days posthatching (Dph) was predominantly composed of the phyla Proteobacteria, Planctomycetes, Firmicutes, and Bacteroidetes [9]. Specifically, Proteobacteria dominate the initial developmental stages, while Planctomycetes become prevalent in subsequent ontogenetic stages, as supported by prior investigations [10].

However, these low-frequency sampling studies have yielded conflicting results, highlighting the need for fine-scale temporal investigations into GFP larval microbiota in aquaculture settings [8]. Importantly, unlike relative abundance (RA)—which profiles the proportional distribution of microbial taxa within communities across time and space—Accurate 16S absolute quantification sequencing focuses on changes in absolute abundance (AA), enabling assessments of whether a species thrives or declines over time [15,16]. Conventional relative abundance-based methods may introduce analytical biases, as observed shifts in community composition could reflect either genuine ecological changes or artifacts arising from fluctuations in host biomass [17]. Thus, absolute quantification sequencing provides a more accurate depiction of community dynamics, facilitating the identification of biotic and abiotic factors shaping microbial communities and strengthening correlations between microbiota characteristics and host traits [18,19]. Meanwhile, the core microbiome is a common feature of microbial communities during biological development [20]. However, defining core microorganisms solely based on “presence/absence” (e.g., considering taxa present in ≥80% of samples as core) typically overlooks the contribution of species abundance to ecological functions [21,22]. In contrast, the abundance–occupancy model quantifies both the abundance and distribution of species across samples, enabling the identification of core species with “high abundance” and “wide distribution”—a definition more aligned with that of “ecological keystone species” [22]. Unlike the core microbiome, the “persistent microbiome” integrates not only the presence of microbial taxa but also their abundance and the influence of stochastic factors [21]. A 2020 study by Liu et al. demonstrated that stochastic processes shape the biogeographical variations in core microbial communities of both aerial and belowground compartments of common beans, confirming the crucial role of stochastic processes in core microbial groups [23].

Understanding the community composition and succession of the larval microbiota could significantly contribute to the success of GFP aquaculture through microbial management strategies [7,8,24]. Thus, we hypothesized that high-frequency sampling would reveal stage-specific microbial dynamics undetectable in prior low-resolution studies. Absolute quantification would correct distortions from host-biomass fluctuations and PCR bias. Larval community assembly would be dominated by stochastic processes, enabling non-antibiotic management. In this study, we employed a novel integrated approach combining high-frequency temporal sampling (covering all eleven zoeal stages and 1 postlarval stage) with Accurate 16S absolute quantification sequencing to (1) resolve stage-specific bacterial dynamics (including absolute abundance of bacteria, richness of observed species, Shannon–Wiener index, phylogenetic diversity, and Pielou’s evenness) and predicted functional potentials in giant freshwater prawn (*GFP*; *Macrobrachium rosenbergii*) larvae at unprecedented temporal resolution; and (2) quantify the ecological processes governing community assembly through neutral modeling and abundance–occupancy relationships, and explore the persistent microbiome.

## 2. Materials and Methods

### 2.1. Experimental Design and Sample Collection

The larval nursery ponds were located at the Nanyu Agricultural Technology Development Co., Ltd., Huzhou, China. Only one ovigerous GFP was used in this study to minimize genetic divergence and inter-individual differences. The newly hatched zoeae were distributed into the six rearing tanks with 1500 larvae per tank. The larval culture was carried out using the identical management model, including pre-treatment of seawater, water exchange, breeding density, and feed schedule, according to Xu et al. [9]. Seawater was aerated constantly and controlled at 28 to 30 °C, salinity of 11 PSU, pH of 8.0 ± 0.1, dissolved oxygen of 8.0 ± 0.2 mg/L, and ammonia nitrogen of <0.1 mg/L throughout the larval nursery. Light intensity was controlled at 1000–1500 lx under a 12:12 h light/dark photoperiod. From 2 days posthatch (Dph), zoeae were fed twice daily with brine shrimp (Artemia) larvae. After completing metamorphosis on Dph 9, larvae were gradually transitioned to an egg yolk diet. Postlarvae underwent gradual desalination. During cultivation, sewage was removed daily, and 1/5–1/4 of the total water volume was replaced.

To ensure the collected larvae were in the same development stage, the newly formed larvae at each stage were sampled only when ≥90% of the population had molted to the target stage. The larval development stage was confirmed via microscopy based on the morphological characteristics described by Zhao et al. (2010) [2]. The zoea and postlarvae were randomly sampled 6 times from each tank and quantified across all developmental stages. The larvae were soaked and washed for 10–15 s with sterilized water to remove the adsorbed rearing water, and then were transferred into sterilized and enzyme-free centrifuge tubes, followed by centrifugation at 27× *g* for 1 min, to obtain larval precipitates (about 0.03 g/tank). A total of 126 GFP larvae samples were collected and stored at −80 °C until DNA extraction. At the same time, temperature, salinity, total ammonia nitrogen (TAN), NO_2_^−^-N, NO_3_^−^-N, DO, pH, and total dissolved solids (TDS) were measured using a water quality flow injection instrument (Jitian Biotechnology Co., Ltd., Beijing, China).

### 2.2. DNA Extraction and Amplicon Sequencing

Accu16S (Accurate 16S absolute quantification sequencing) was performed by Genesky Biotechnologies Inc., Shanghai, 201315 (China). Briefly, total genomic DNA was extracted using the FastDNA SPIN Kit for Soil (MP Biomedicals, Santa Ana, CA, USA) according to the manufacturer’s instructions. The integrity of genomic DNA was detected through agarose gel electrophoresis, and the concentration and purity of genomic DNA were detected through the Nanodrop 2000 and Qubit 3.0 Spectrophotometer (Thermo Fisher Scientific, Waltham, MA, USA). Multiple spike-ins with identical conserved regions to natural 16S rRNA genes and variable regions replaced by random sequence with ~40% GC content were artificially synthesized. Then, the appropriate proportions of a spike-in mixture with known gradient copy numbers was added to the sample DNA. The V3–V4 hypervariable regions of the 16S rRNA gene and spike-in were amplified with the primers 341F (5′-CCTACGGGNGGCWGCAG-3′) and 805R (5′-GACTACHVGGGTATCTAATCC-3′) and then sequenced using an Illumina NovaSeq 6000 sequencer (Illumina, San Diego, CA, USA).

### 2.3. Illumina Read Data Processing and Analysis

The raw read sequences were processed in QIIME2. The adaptor and primer sequences were trimmed using the cutadapt plugin. The DADA2 plugin was used for quality control and to identify amplicon sequence variants (ASVs). Taxonomic assignments of ASV representative sequences were performed with a confidence threshold of 0.7 via a pretrained Naive Bayes classifier, which was trained on the SILVA (version 138.2) [25,26]. Then, the spike-in sequences were identified, and reads were counted. The standard curve for each sample was generated based on the read-counts versus spike-in copy number, and the absolute copy number of each ASV in each sample was calculated by using the read-counts of the corresponding ASV. Since the spike-in sequence is not a component of the sample flora, the spike-in sequence needs to be removed in the subsequent analysis. The original MiSeq 16S rRNA sequence data generated in this study have been deposited in the Sequence Read Archive under the accession number NMDC10019917.

### 2.4. Data Analyses

All bioinformatic analysis was conducted in the R v4.0.0 project [27]. The α-diversity, including the Shannon–Wiener index, richness of observed species, and phylogenetic diversity, of each sample was computed using QIIME2 [26]. Pielou’s evenness was calculated via the “vegan” R package v2.5-3 [27]. To visualize the overall taxonomic and phylogenetic turnover of the larval, principal coordinate analysis (PCoA) was performed. The time decay of the community similarity relationship was plotted as the logarithmic similarity against logarithmic age, and a linear regression was performed to obtain the temporal turnover rate [8]. The relationship between the Bray–Curtis similarity and the temporal gradient was assessed by plotting the Bray–Curtis similarity against the gradient variable and fitting a linear regression model to quantify the rate of community compositional change. Persistent microbiomes were identified based on the abundance and occupancy distribution by Stopnisek and Shade [21,22]. Firstly, taxa should be ranked based on occupancy, and optionally, weighted in conjunction with taxon abundance. The contribution of the core taxon subset to β-diversity was quantified by computing the proportion of community similarity attributed to the core taxa. To pinpoint the inflection point where the marginal gain in the explanatory value plateaus as the core inclusion threshold escalates, this study adopts a more rigorous “elbow method” grounded in first-order differences. For each dataset, the cumulative explanatory value of incorporating the subsequent taxon into the core set was calculated. Finally, the neutral model was utilized to deduce whether the assembly of core taxa is deterministic. The PICRUSt2 (Phylogenetic Investigation of Communities by Reconstruction of Observed States, v2.1.0-b) pipeline was used to infer functional potentials of larval GFP microbiota [28]. All visualizations were generated using the “ggplot2” v3.4.2 [24] and “patchwork” v1.1.2 R packages.

## 3. Results

### 3.1. Abundance and Alpha-Diversity of Bacterial Community

**Figure 1:**
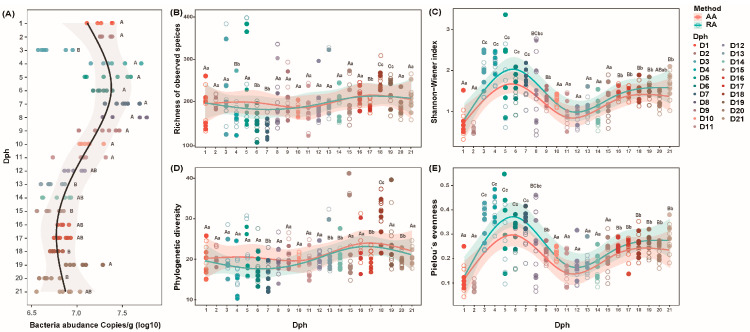
Abundance and diversity changes in the quantitative GFP larvae bacterial community (**A**) Absolute abundance dynamics of bacteria. (**B**) Richness of observed species of the samples during the 21 Dph. (**C**) Shannon–Wiener index of the samples during the 21 Dph. (**D**) Phylogenetic diversity of the samples during the 21 Dph. (**E**) Pielou’s evenness of the samples during the 21 Dph. DPH, days posthatch. The different letters indicate a significant difference between stages (*p* < 0.05) (uppercase for AA; lowercase for RA).

To investigate the developmental trajectory of the microbial community in the GFP larvae throughout the zoea and postlarvae period (1–21 days of age), we examined 126 samples using the absolute quantification sequencing method (Figure S1). We found that the AA of bacteria exhibited a phased pattern of “initial accumulation, mid-stage expansion, and late-stage remodeling” during larval development (Figure 1).

Specifically, during the initial colonization stage (Dph 1 to 5), the absolute abundance (AA) of bacteria increased linearly during early development, while in the mid-stage expansion phase (Dph 6 to 15), the AA peaked at (Copies/g _(log10)_ = 7.0–7.5) around Dph 7 to 8. Subsequently, during the decline period (Dph = 8 to 15), the AA gradually decreased after reaching the peak and then tended to stabilize. In the late-stage remodeling phase (Dph 16 to 21), the AA of bacteria increased again, with significant peaks reappearing on the Dph 19 and Dph 21 (Copies_(log10)_ = 7.1, Copies_(log10)_ = 6.8, respectively) (Figure 1A). Similarly, the Shannon index and Pielou’s evenness of larvae were at a high level from Dph 6–8, subsequently decreased during Dph 11 to 13, and finally returned to the previous high level and remained stable from Dph 16 to 21. However, the richness of observed species and phylogenetic diversity remained relatively stable during the ontogenetic trajectory of GFP larvae. There were only rapid fluctuations in the richness of observed species and phylogenetic diversity from Dph 6 to 14, but these indices immediately regained stability afterward.

### 3.2. Dynamics of Bacterial Composition of GFP Larvae

**Figure 2:**
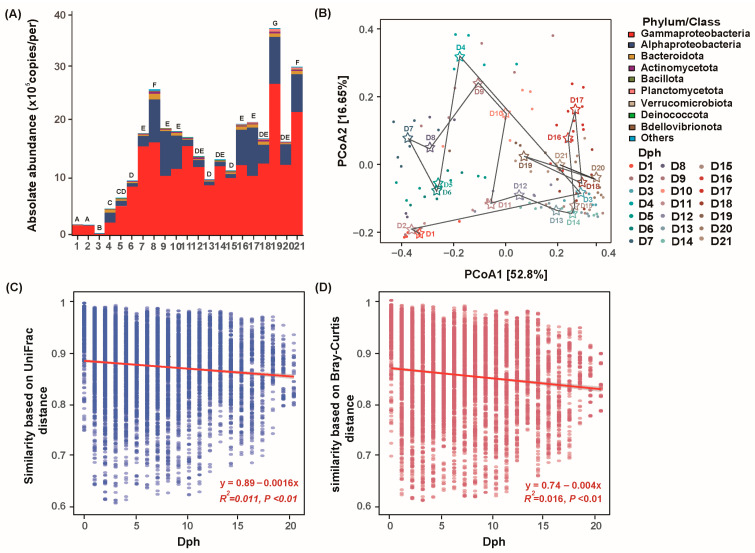
Dynamics of GFP larval bacterial communities. (**A**) Changes in the absolute abundance of dominant phyla/proteobacterial classes (Top 10) of GFP larval bacterial communities. (**B**) Principal coordinate analysis (PCoA) visualizing compositional variations in absolute abundance of larval bacterial communities across developmental stages based on Bray–Curtis dissimilarity. (**C**) Phylogenetic similarity based on UniFrac distance (1—weighted UniFrac distance). (**D**) Time decay in similarity between larval bacterial communities based on Bray–Curtis similarity. Dph, days posthatch. The different letters indicate a significant difference between stages (*p* < 0.05). Different colored stars indicate different developmental stages.

During the ontogenetic trajectory of GFP larvae, the bacterial communities were mainly dominated by γ-Proteobacteria, α-Proteobacteria, Bacteroidota, Actinomycetota, Bacillota, Planctomycetota, Verrucomicrobiota, Deinococcota, and Bdellovibrionota (Figure 2A and Figure S2). Furthermore, at the individual level, the results of both relative and absolute quantification showed that γ-Proteobacteria and α-Proteobacteria were the commensals with the highest abundance (Figure 2A). Delving deeper into the developmental dynamics, the bacterial community composition and abundance exhibited distinct temporal patterns. In detail, during the Zoea I-Zoea V (Dph 1 to 6), γ-Proteobacteria dominated absolutely, with an RA level of over 80% and an absolute abundance ranging from 1.9 × 10^5^ to 8.7 × 10^5^ copies per sample (Figure S3). On the Dph 8 (Zoea VI), the abundance of γ-Proteobacteria increased rapidly and remained at a high level in all subsequent samples, with an absolute abundance consistently higher than 22.8 × 10^5^ copies per sample, highlighting its persistent prevalence throughout the development process. Specifically, it peaked on Dph 19 (Zoea XI) and Dph 21(Postlarvae), reaching 68.9 × 10^5^ copies per sample and 52.3 × 10^5^ copies per sample, respectively. Meanwhile, α-Proteobacteria showed a significant upward trend over time but remained distinctly subordinate to γ-Proteobacteria in the community. In detail, the abundance of α-Proteobacteria began to rise on the Dph 4 (Zoea IV), with an absolute abundance of 1.8 × 10^5^ copies per sample. It gradually became a dominant group in samples Dph 8 to 11, with an absolute abundance reaching 15.1–19.4 × 10^5^ copies per sample. Then, from the Dph 11 to 15 (ZoeaVII to ZoeaIX), its abundance dropped back to 1.7–3.1 × 10^5^ copies per sample, but it rebounded on Dph16, once again becoming a dominant group with an absolute abundance of 21.9 × 10^5^ copies per sample. In addition, in contrast to the results of relative quantification, the absolute abundances of other major phyla, such as Actinomycetota, Bacillota, and Planctomycetota, started to increase from the third day and remained consistently at the level of approximately 1.5 × 10^5^ copies per sample thereafter (Figure 2A).

### 3.3. Taxonomic and Phylogenetic Turnover of Bacterial Community with GFP Larval Development

In general, the bacterial community composition of GFP larvae was clustered according to developmental stages (Figure 2B). The taxonomic composition of the bacterial community based on AA in larvae showed distinct successional trajectories, while the taxonomic composition of the bacterial community based on RA overlapped to some extent during the sub-stages of the zoea stage (Figure S2B). As evidenced via one-way analysis of similarity (ANOSIM), the compositions of larval communities were significantly different at all stage (all *p* < 0.01).

Furthermore, the succession of larval bacterial communities taxonomically and phylogenetically fitted the time decay model (all *p* < 0.01). Crucially, ANCOVA confirmed that the phylogenetic turnover rate (w = −0.89, R^2^ = 0.011) was significantly steeper than then ASV turnover (w = −0.74, R^2^ = 0.016) (F = 9.52, *p* < 0.001) (Figure 2C,D). This indicates larval microbiota resisted environmental disturbances via phylogenetic replacement (substituting phylogenetically similar taxa) rather than species replacement, preserving functional stability despite compositional shifts.

### 3.4. Persistent Microbiome Is Detected Across GFP Larval Development

**Figure 3:**
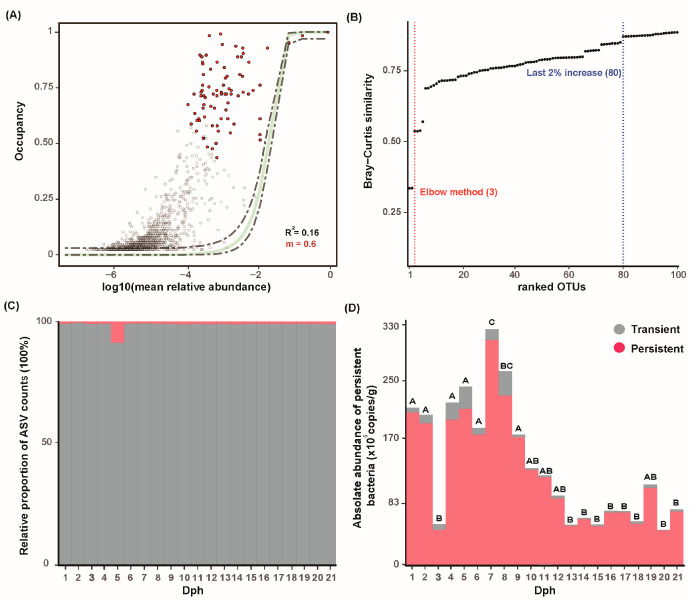
A persistent microbiome is detected across GFP larval development. (**A**) Abundance–occupancy distributions were used to identify persistent members. A neutral model of community assembly fit to an abundance–occupancy distribution. Each point is a different ASV, and the persistent members are red points while non-core taxa are gray points. Each point is an ASV plotted by its mean log_10_ absolute abundance and occupancy, the solid green line is the neutral model, and the dashed gray lines are 95% confidence intervals around the model fit. The points that fall outside the 95% model confidence are inferred to be deterministically, rather than neutrally, selected. Points above the model are selected by the environment, and points below the model are dispersal-limited. (**B**) Bray–Curtis similarity across ranked OTUs: Elbow method and last 2% increase identification. (**C**) The number of ASVs of different occurrence frequencies (persistent and transient) is shown. (**D**) The absolute abundance of ASVs of different occurrence frequencies (persistent and transient) is shown. The different letters indicate a significant difference between stages (*p* < 0.05).

Based on abundance–occupancy calculations, 80 ASVs were identified as persistent microbiota during the larval development of GFP larvae (Figure 3A,B). In detail, there is 1 ASV belonging to Deinococcota; 2 ASVs (ASV115 and ASV117) belonging to Bacillota; 2 ASVs (ASV72 and ASV85) belonging to Planctomycetota; six ASVs (ASV26, ASV48, ASV49, ASV62, ASV120, and ASV186) belonging to Actinomycetota; 13 ASVs (ASV4, ASV16, ASV17, ASV20, ASV31, ASV38, ASV60, ASV63, ASV64, ASV68, ASV76, ASV125, and ASV134) belonging to Bacteroidota; 26 ASVs (ASV2, ASV18, ASV19, ASV21, ASV24, ASV25, ASV29, ASV32, ASV33, ASV35, ASV40, ASV41, ASV43, ASV44, ASV50, ASV53, ASV57, ASV58, ASV59, ASV75, ASV96, ASV97, ASV102, ASV119, ASV141, and ASV142) belonging to Alphaproteobacteria; and 30 ASVs (ASV1, ASV3, ASV6, ASV7, ASV8, ASV10, ASV14, ASV15, ASV23, ASV28, ASV30, ASV42, ASV46, ASV52, ASV54, ASV61, ASV79, ASV83, ASV90, ASV101, ASV104, ASV106, ASV107, ASV111, ASV112, ASV113, ASV116, ASV124, ASV135, and ASV155) belonging to Gammaproteobacteria. The persistent bacterial ASVs possessed < 1% of the total ASVs but accounted for more than 95% of bacterial AA, and they were mainly composed of the following: *genus: Herminiimonas*, *genus: Maritalea*, family: Enterobacteriaceae; *genus: Flavobacterium*, family: Alcaligenaceae; and family: Paracoccaceae (Figure 3C,D). In addition, the assembly of larval bacteria was predominantly governed by neutral processes, and the neutral model (R^2^ = 0.16, m = 0.6) performed better than the binomial distribution model (according to Akaike Information Criterion, AIC) (Figure 3A). In particular, ASV1 (*genus: Herminiimonas*), ASV2 (*genus: Maritalea*), and ASV3 (family: Enterobacteriaceae) conformed to the neutral distribution.

### 3.5. Functional Potential of GFP Larval Bacterial Community and Change Patterns

**Figure 4:**
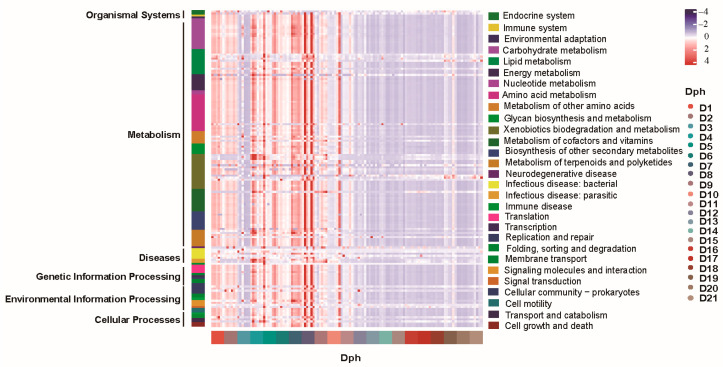
Functional potential of GFP larval bacterial community. Dph, days posthatch.

To reveal the changes in the functional potential of the GFP larval bacterial community, we first used the PICRUSt2 tool to predict the functional potentials (Figure 4). The results showed significant differences in the functional profiles of seawater bacterial communities between pre- and post-feeding with egg yolk. In general, functional potentials relevant to organismal systems, genetic information processing, environmental information processing, cellular processes, and metabolism-relevant potentials were enriched in larvae pre-feeding with egg yolk (Dph < 9) compared to larvae at post-feeding with egg yolk (Dph 10 to 21) (Table S1). These results imply that the changed bacterial community, with its altered functional potentials, may play vital roles in meeting the changing nutritional and physiological demands during the development of GFP larvae.

## 4. Discussion

Our findings reveal that bacterial colonization is inherently tied to host ontogeny: As the larval gut structure becomes more complex, the range of available ecological niches expands. This supports the effectiveness of environmental optimization over disruptive intervention strategies. The absolute abundance of bacteria throughout larval development follows a “three-phase trajectory”—initial accumulation, mid-stage expansion, and late-stage remodeling—refining and extending conclusions from previous research. Similar to the larval stages of shrimp and zebrafish, the taxonomic and phylogenetic composition of the bacterial community in giant freshwater prawn (GFP) larvae exhibits distinct stage-specific patterns [7,8,29]. Absolute quantification data show that individual bacterial absolute abundance (AA) increases during larval development, mirroring the trend in α-diversity.

In the early larval stages, the microbial community is in an initial establishment phase. The host’s physiological functions are underdeveloped, and the intestinal structure remains simple, offering limited ecological niches that can only support colonization by a small number of highly adaptable bacterial groups [30,31]. Notably, the bacterial community begins to form when zoeae start feeding at the second stage [32]. Concurrently, larval molting promotes the flushing and reassembly of epidermal bacterial communities, resulting in lowα-diversity and bacterial abundance [8,33]. As the host matures, dietary shifts introduce new substrates and selective pressures on the microbial community [34,35]. Meanwhile, the maturation of the GFP’s intestinal tract and gradual improvement of immune function expand ecological niches, leading to a significant increase in bacterial abundance [36,37]. Additionally, previous studies have noted that during the mid-zoeal stage (7 Dph), interactions between water and larval bacterial communities cause fluctuations in α-diversity [9,35]. As larvae continue to mature, the intestinal tract and other organs become more developed, providing stable and diverse ecological niches [33,38], facilitating the colonization and survival of a broader range of microorganisms, and eventually stabilizing α-diversity [5,6].

At the ASV level, shifts in physiological status, dietary patterns, and microbial sources before and after the dietary transition may drive significant changes in the larval bacterial community [8,24,35]. Notably, after being fed egg yolk, the ASVs belonging to the Alphaproteobacteriahe bacterial community changed rapidly. However, the temporal turnover model indicated that the ASV turnover rate of the larval bacterial community was lower than the phylogenetic turnover rate, suggesting that the larval microbiota resisted environmental disturbances through “phylogenetic replacement rather than species replacement” to maintain functional stability [39,40]. This result supports the theory of “phylogenetic community ecology” that is, community structure is determined not only by the current species composition but also profoundly influenced by evolution [41,42]. This implies that the larval bacterial community may rapidly adapt to the host’s developmental needs by preferentially retaining phylogenetically related species. Interestingly, PICRUSt2 prediction analysis further showed that during the early developmental stage (from first feeding to day 9), the metabolic potential enrichment of various organic matters in the shrimp larval microbiota was consistent with the predicted results. Similar trends have often been reported in the larval stages of *Penaeus vannamei* and *Macrobrachium nipponense* [8,9,43]. Given their immature digestive systems, GFP larvae may partially rely on bacteria for food digestion and nutrient metabolism [3,4,35]. Thus, the high variability in bacterial community composition is driven by the stage-specific physiological demands of the host [7,39]. However, the active functions of these bacteria were not assessed in the present study, warranting further investigation. In future research, we will integrate targeted metabolomics (such as quantitative lipidomics) and metatranscriptomics to directly verify the active expression of key metabolic pathways.

The absolute quantification results showed that Gammaproteobacteria and Alphaproteobacteria remained absolutely dominant throughout the larval development process. Notably, this conclusion is inconsistent with the view proposed by Xu et al. [9]. In contrast, it is highly consistent with those of Ma et al. and Liu et al. [10,35]. The bacterial abundance would fluctuate with larval developmental stages (especially the dietary transition from Artemia to egg yolk) and organ maturation, and the observed peak at Dph 7–8 (Zoea VI, early stage of dietary transition) aligns with this expectation. Compared with Xu et al. (2022) [9], our absolute abundance peak (log10 = 7.0–7.5 copies/g) is higher, which may be due to the bias of relative quantification methods in reflecting real microbial proliferation [17], while Accu16S provides more accurate data. Consistent with Ma et al. (2020) [10], both studies confirmed the dominance of Gammaproteobacteria during larval development, supporting the core role of this group in larval microbiota. Compared to Wang et al. (2020) [8] who studied on *P. vannamei* larvae, both studies observed a “rise-then-stable” trend in α-diversity, but our high-frequency sampling captured a transient decline at Dph 11–13 (possibly related to molting), which was not detected in low-frequency sampling studies. These comparisons highlight the advantages of our high-frequency sampling and absolute quantification methods in revealing fine-scale microbial dynamics, while confirming the generality of the key findings (e.g., dominance of Gammaproteobacteria) across crustacean larval studies. This deviation could be attributed to multiple factors, such as sequencing methods, the choice of DNA extraction kits (which might lead to DNA recovery bias), sampling methods, and detection methods [44]. Traditional approaches to defining the core microbiome, based solely on abundance or presence/absence, have limitations: abundance-based methods may overlook important low-abundance taxa, while presence/absence-based methods may include transient, ecologically insignificant taxa [22]. “Persistence” supersedes traditional “core microbiome” definitions, identifying keystone taxa that maintain functional resilience via niche partitioning. After high-frequency sampling, the abundance occupancy model can effectively avoid this problem and more accurately identify 80 persistent ASVs that account for 95% community abundance during the larval development of GFP. Notably, ASV1 (*Herminiimonas*), ASV2 (*Maritalea*), and ASV3 (Enterobacteriaceae) maintain absolute dominance, with their abundance primarily shaped by stochastic processes rather than deterministic environmental selection. This suggests that environmental factors have a minimal impact on their abundance, and the community structure is mainly maintained by species dispersal capacity and ecological drift [45,46]. The genus *Herminiimonas* can decompose organic debris (such as uneaten feed and feces) and plays a stable role in the carbon cycle [47]. It also secretes digestive enzymes to aid larval digestion of animal feed, improving nutrient absorption efficiency. Additionally, *Herminiimonas* may supply minerals through metabolic activities, facilitating exoskeleton formation during molting [48]. *Maritalea* can degrade complex organics via genome-encoded hydrolases. It may colonize the GFP’s body surface or intestine through random diffusion, and its metabolic traits allow it to maintain abundance across varying salinities [11]. *Maritalea* may form symbiotic relationships with marine invertebrates by supplying nutrients (e.g., synthesizing vitamins/amino acids) or degrading host metabolic waste. Enterobacteriaceae are key components of microbial communities in shrimp intestines, body surfaces, and gills across developmental stages [44]. Their proliferation was promoted by the low-salinity, eutrophic, and high-density larval rearing environment of GFP, with colonization achieved via the food chain or water contact. Digestive enzymes (e.g., proteases, amylases, and lipases) were secreted by Enterobacteriaceae to facilitate the breakdown of dietary proteins, carbohydrates, and lipids, with feed digestion and nutrient utilization in shrimp enhanced accordingly [9,10,12]. However, as opportunistic pathogens, they can transition to a pathogenic state could be made by them under stress (e.g., poor water quality, temperature fluctuations, and high-density farming) or immune suppression; during this time, the intestinal barrier can be breached, potentially allowing tissue invasion and causing infectious diseases [12]. Coexistence might be achieved by the three major ASVs through niche partitioning: the posterior part of the shrimp intestine might be colonized by Herminiimonas to utilize undigested fibers of the host, the mucus layer on the body surface might be preferred by *Maritalea* to utilize organic matter in water, and the anterior part of the intestine might be dominated by Enterobacteriaceae through facultative anaerobic metabolism. This spatial differentiation reduces interspecific competition, making stochastic processes the primary driver of abundance changes [29,30].

Fitting the neutral model allowed for the quantification of dispersal limitation intensity for the three dominant ASVs, revealing that stochasticity outweighs deterministic selection-supporting microbiome management through environmental modulation [5,45]. In this study, an explanatory R^2^ = 0.16 was obtained by the neutral model, indicating the feasibility of exogenous regulation of the larval bacterial communities [24]. Although stable abundances were achieved by the dominant groups under the neutral distribution, their functional expressions might fluctuate with the environment [29]. A high abundance was maintained by Enterobacteriaceae under the neutral model, but the proportion of toxin-producing strains might fluctuate due to random drift, increasing the pathogenic risk to the host. If the three major ASVs conformed to the neutral distribution, blind application of antibiotics or probiotics could be reduced, and instead, the aquaculture environment could be optimized (such as controlling water transparency and dissolved oxygen) to decrease the promotion of random drift on the proliferation of harmful bacteria. Based on the results of absolute quantification and high-frequency sampling, the study suggests that in the larviculture of *M. rosenbergii*, when replacing Artemia with egg yolk at specific larval stages (such as the late Zoea stage), high-abundance functional microbial communities should be introduced simultaneously. This measure can match the larvae’s peak nutritional demand and avoid growth stagnation caused by nutritional supply gaps. Strains of Herminiimonas with dual functions can be developed as probiotics to reduce larval mortality during the dietary transition period. Neutral model analysis shows that microbial community assembly is dominated by ecological drift (correlation coefficient R^2^ ≈ 0.16), indicating that regulating environmental stochasticity can reduce the promotion of pathogenic bacteria by deterministic selection pressure. It is possible to reduce potential disease risk and significantly lower mortality without relying on antibiotics through the addition of probiotics in feed and the colonization of beneficial bacteria in water bodies. Future studies are therefore recommended to validate these results using multiple broodstock individuals, which would enhance the generalizability of the conclusions and provide a more comprehensive understanding of larval microbiome assembly across different genetic backgrounds.

## 5. Conclusions

This study, using high-frequency sampling combined with 16S rRNA gene absolute quantification, revealed the dynamic patterns of microbial communities during larval development of *Macrobrachium rosenbergii*. The bacterial absolute abundance followed a triphasic trajectory. Bacterial colonization was intrinsically associated with host ontogeny; as the complexity of the larval gut increased, the availability of ecological niches expanded, validating the effectiveness of environmental optimization over disruptive interventions. Eighty persistent Amplicon Sequence Variants (ASVs) accounted for over 95% of the community abundance. Neutral model analysis indicated that the abundances of three dominant ASVs (*Herminiimonas*, *Maritalea*, and Enterobacteriaceae) were driven by ecological drift and dispersal limitation rather than deterministic selection.

The early-stage microbiota was enriched in organismal systems, metabolism, and information processing functions, which precisely corresponded to the dietary shift from Artemia to egg yolk. The metabolic plasticity of the microbiota buffered nutritional transitions; however, under neutral drift, opportunistic pathogens required continuous monitoring. This work confirmed that phylogenetic replacement was the core strategy for the microbiota to resist environmental disturbances, with stochastic processes dominating the early community assembly.

These findings provided a theoretical basis for optimizing aquaculture environments instead of relying on antibiotics. They also offered crucial insights into early life microbe–host interactions and niche differentiation in aquatic animals. Mechanistically, future research could resolve the keystone functions of persistent taxa via metatranscriptomics. To validate neutral model predictions in commercial hatcheries, it would be necessary to manipulate dispersal vectors. Developing Enterobacteriaceae-suppressing probiotics that exploit niche competition without disturbing stochastically assembled communities is also a promising direction. Moreover, integrating host transcriptomics could disentangle microbe-mediated development cues from environmental stochasticity.

## Data Availability

The data presented in this study are openly available in [Quantitative Analysis of 16S rRNA Genes in the Gut Microbiota During Larval Development of Macrobrachium rosenbergii] [https://nmdc.cn/resource/genomics/project/detail/NMDC10019917 (accessed on 25 June 2025)] [NMDC10019917].

## Supplementary Materials

The following supporting information can be downloaded at: https://www.mdpi.com/article/10.3390/microorganisms13081881/s1, Figure S1: Experimental design and sampling schedules; Figure S2: (A) Changes in the relative abundance of dominant phyla/proteobacterial classes (Top 10) of GFP larval bacterial communities. (B) Principal coordinate analysis (PCoA) visualizing compositional variations of larval bacterial communities across developmental stages based on Bray-Curtis dissimilarity. Figure S3: Analysis of microbial ANOSIM at each larval developmental stage of Macrobrachium rosenbergii. Table S1: Functional potentials between pre-feeding with egg yolk (Dph < 9) compared with larvae at post-feeding with egg yolk (Dph 10 to 21).

## References

[1] Pillai B.R., Ponzoni R.W., Das Mahapatra K., Panda D. (2022). Genetic improvement of giant freshwater prawn 638 *Macrobrachium rosenbergii*: A review of global status. Rev. Aquac..

[2] Zhao C., Zhou X., Yuan X., Xi Q. (2010). Morphological characteristics of zoaea larva of *Macrobrachium rosenbergii*. J. Guangdong Ocean Univ..

[3] Vadstein O., Attramadal K.J.K., Bakke I., Olsen Y. (2018). K-selection as microbial community management strategy: A method for improved viability of larvae in aquaculture. Front. Microbiol..

[4] Vestrum R.I., Attramadal K.J.K., PER W., Li K., Olsen Y., Bones A.M., Vasdtein O., Bakke I. (2018). Rearing water treatment induces microbial selection influencing the microbiota and pathogen associated transcripts of cod (*Gadus morhua*) larvae. Front. Microbiol..

[5] Xiong J., Wang K., Wu J., Qiuqian L., Yang K., Qian Y., Zhang D. (2015). Changes in intestinal bacterial communities are closely associated with shrimp disease severity. Appl. Microbiol. Biotechnol..

[6] Xiong J., Zhu J., Dai W., Dong C., Qiu Q., Li C. (2017). Integrating gut microbiota immaturity and disease-discriminatory taxa to diagnose the initiation and severity of shrimp disease. Environ. Microbiol..

[7] Xiong J., Li X., Yan M., Lu J., Qiu Q., Chen J. (2020). Comparable ecological processes govern the temporal succession of gut bacteria and microeukaryotes as shrimp aged. Microb. Ecol..

[8] Wang Y., Wang K., Huang L., Dong P., Wang S., Chen H., Zhang D. (2020). Fine-scale succession patterns and assembly mechanisms of bacterial community of *Litopenaeus vannamei* larvae across the developmental cycle. Microbiome.

[9] Xu Y., Cheng H., Gu Z., Li X., Shen P., Gao Q. (2022). Study on microflora structure of *Macrobrachium rosenbergii* larvae and environmental water at different developmental stages. J. Freshw. Ecol..

[10] Ma R., Wang Y., Zhao S., Yin M., Fang W. (2020). The composition of the microbial community associated with *Macrobrachium rosenbergii* zoeae varies throughout larval development. J. Fish. Dis..

[11] Shirsalimian M.S., Akhavan S.A., Dabbagh A.M.A.R. (2018). Isolation of two radiation resistant and desiccation tolerant bacteria, *Modestobacter* sp. *A2* and *Maritalea* sp. *B9*, from gandom beryan hill in the lut desert of Iran. Microbiology.

[12] Gao X., Zhou Y., Zhu X., Tang H., Li X., Jiang Q., Wei W., Zhang X. (2021). *Enterobacter cloacae*: A probable etiological agent associated with slow growth in the giant freshwater prawn *Macrobrachium rosenbergii*. Aquaculture.

[13] Li C., Jin L., Zhang C., Li S., Zhou T., Hua Z., Wang L., Ji S., Wang Y., Gan Y. (2023). Destabilized microbial networks with distinct performances of abundant and rare biospheres in maintaining networks under increasing salinity stress. iMeta.

[14] Zhang L., Xu S., Zhang Z., Zhang X., Liu X. (2023). Transcriptomic profiling and characterization of microRNAs in *Macrobrachium rosenbergii* potentially involved in immune response to *Enterobacter cloacae* infection. Microb. Pathog..

[15] Rao C., Coyte K.Z., Bainter W., Geha R.S., Martin C.R., Rakoff-Nahoum S. (2021). Multi-kingdom ecological drivers of microbiota assembly in preterm infants. Nature.

[16] Zhang J., Liu Y., Zhang N., Hu B., Jin T., Xu H., Qin Y., Yan P., Zhang X., Guo X. (2019). NRT1.1B is associated with root microbiota composition and nitrogen use in field-grown rice. Nat. Biotechnol..

[17] Feng Y., Zhang M., Liu Y., Yang X., Wei F., Jin X., Liu D., Guo Y., Hu Y. (2023). Quantitative microbiome profiling reveals the developmental trajectory of the chicken gut microbiota and its connection to host metabolism. iMeta.

[18] Qiao Y., Wang Z., Sun H., Guo H., Song Y., Zhang H., Ruan Y., Xu Q., Huang Q., Shen Q. (2024). Synthetic community derived from grafted watermelon rhizosphere provides protection for ungrafted watermelon against *Fusarium oxysporum* via microbial synergistic effects. Microbiome.

[19] Li S., Delgado-Baquerizo M., Ding J., Hu H., Huang W., Sun Y., Ni H., Kuang Y., Yuan M.M., Zhou J. (2024). Intrinsic microbial temperature sensitivity and soil organic carbon decomposition in response to climate change. Glob. Change Biol..

[20] Yajima D., Fujita H., Hayashi I., Shima G., Suzuki K., Toju H. (2023). Core species and interactions prominent in fish-associated microbiome dynamics. Microbiome.

[21] Stopnisek N., Shade A. (2021). Persistent microbiome members in the common bean rhizosphere: An integrated analysis of space, time, and plant genotype. ISME J..

[22] Shade A., Stopnisek N. (2019). Abundance-occupancy distributions to prioritize plant core microbiome membership. Curr. Opin. Microbiol..

[23] Liu Y., Li D., Qi J., Peng Z., Chen W., Wei G., Jiao S. (2021). Stochastic processes shape the biogeographic variations in core bacterial communities between aerial and belowground compartments of common bean. Environ. Microbiol..

[24] Lu Z., Ren Z., Lin W., Shi C., Mu C., Wang C., Wu Q., Ye Y. (2022). Succession, sources, and assembly of bacterial community in the developing crab larval microbiome. Aquaculture.

[25] DeSantis T., Hugenholtz P., Larsen N., Rojas M., Brodie E., Keller K., Huber T., Dalevi D., Hu P., Andersen G.L. (2006). Greengenes, a chimera-checked 16S rRNA gene database and workbench compatible with ARB. Appl. Environ. Microbiol..

[26] Edgar R. (2010). Search and clustering orders of magnitude faster than BLAST. Bioinformatics.

[27] Oksanen J., Blanchet F.G., Friendly M., Kindt R., Legendre P., McGlinn D., Minchin P.R., O’Hara R.B., Simpson G.L., Solymos P. (2018). Vegan: Community Ecology Package, R package version 2.5-3.

[28] Douglas G.M., Maffei V.J., Zaneveld J.R., Yurgel S.N., Brown J.R., Taylor C.M., Huttenhower C., Langille M.G.I. (2020). PICRUSt2 for prediction of metagenome functions. Nat. Biotechnol..

[29] Burns A.R., Stephens W.Z., Stagaman K., Wong S., Rawls J.F., Guillemin K., Bohannan B.J.M. (2016). Contribution of neutral processes to the assembly of gut microbial communities in the zebrafish over host development. ISME J..

[30] Xiong J., Dai W., Qiu Q., Zhu J., Yang W., Li C. (2018). Response of host-bacterial colonization in shrimp to developmental stage, environment and disease. Microb. Ecol..

[31] Gundersen M.S., Vadstein O., De Schryver P., Attramadal K.J.K. (2022). Aquaculture rearing systems induce no legacy effects in Atlantic cod larvae or their rearing water bacterial communities. Sci. Rep..

[32] Tang Y., Wang R., Tan L., Guo L., Duan Y., Yang L., Jiang S., Zhou F., Jiang S., Huang J. (2020). Effects of live microalgae and algae powder on microbial community, survival, metamorphosis and digestive enzyme activity of *Penaeus monodon* larvae at different growth stages. Aquaculture.

[33] Estefanía G.V., Marcel M.P., Kadiya C., Francisco V.A., Teresa G.G., Luis M.C. (2020). Taxonomic and functional changes in the microbiota of the white shrimp (*Litopenaeus vannamei*) associated with postlarval ontogenetic development. Aquaculture.

[34] Kumar T.S., Vidya R., Kumar S., Alavandi S.V., Vijayan K.K. (2017). Zoea-2 syndrome of *Penaeus vannamei* in shrimp hatcheries. Aquaculture.

[35] Liu B., Song C., Gao Q., Liu B., Zhou Q., Sun C., Zhang H., Liu M., Tadese W.A. (2021). Maternal and environmental microbes dominate offspring microbial colonization in the giant freshwater prawn *Macrobrachium rosenbergii*. Sci. Total Environ..

[36] Mente E., Gannon A.T., Nikouli E., Hammer H., Kormas K.A. (2016). Gut microbial communities associated with the molting stages of the giant freshwater prawn *Macrobrachium rosenbergii*. Aquaculture.

[37] Xiong J., Xuan L., Yu W., Zhu J., Qiu Q., Chen J. (2019). Spatiotemporal successions of shrimp gut microbial colonization: High consistency despite distinct species pool. Environ. Microbiol..

[38] Lan X., Peng X., Du T., Xia Z., Gao Q., Tang Q., Yi S., Yang G. (2023). Alterations of the gut microbiota and metabolomics associated with the different growth performances of *Macrobrachium rosenbergii* families. Animals.

[39] Yan Q., van der Gast C.J., Yu Y. (2012). Bacterial community assembly and turnover within the intestines of developing zebrafish. PLoS ONE.

[40] Li E., Xu C., Wang X., Wang S., Zhao Q., Zhang M., Qin J., Chen L. (2018). Gut microbiota and its modulation for healthy farming of Pacific white shrimp *Litopenaeus vannamei*. Rev. Fish. Sci. Aquac..

[41] Ricotta C., Podani J., Schmera D., Bacaro G., Maccherini S., Pavoine S. (2023). The ternary diagram of functional diversity. Methods Ecol. Evol..

[42] Davies T.J., Urban M.C., Rayfield B., Cadotte M.W., Peres-Neto P.R. (2016). Deconstructing the relationships between phylogenetic diversity and ecology: A case study on ecosystem functioning. Ecology.

[43] Tzeng T.D., Pao Y., Chen P., Weng F., Jean W., Wang D. (2015). Effects of host phylogeny and habitats on gut microbiomes of oriental river prawn (*Macrobrachium nipponense*). PLoS ONE.

[44] Zheng Y., Yu M., Liu J., Qiao Y., Wang L., Li Z., Zhang X., Yu M. (2017). Bacterial community associated with healthy and diseased Pacific white shrimp (*Litopenaeus vannamei*) larvae and rearing water across different growth stages. Front. Microbiol..

[45] Zhou J., Ning D. (2017). Stochastic community assembly: Does it matter in microbial ecology?. Microbiol. Mol. Biol. Rev..

[46] Dini-Andreote F., Stegen J., Elsas J., Salles J. (2015). Disentangling mechanisms that mediate the balance between stochastic and deterministic processes in microbial succession. Proc. Natl. Acad. Sci. USA.

[47] Fernandes C., Rainey F.A., Fernanda N.M., Pinhal I., Folhas F., Costa M.S. (2005). *Herminiimonas fonticola* gen. nov., sp. nov., a Betaproteobacterium isolated from a source of bottled mineral water. Syst. Appl. Microbiol..

[48] Dopson M., Johnson D.B. (2012). Biodiversity, metabolism and applications of acidophilic sulfur-metabolizing microorganism. Environ. Microbiol..

