# Supplementary material for: Dynamics and Assembly Mechanisms of Bacterial Communities During Larval Development of Macrobrachium rosenbergii: A High-Frequency Sampling Study Based on 16S rRNA Absolute Quantification Sequencing
Source: Microorganisms. 2025 Aug 12;13(8):1881. doi: 10.3390/microorganisms13081881 (PMC12388580; doi:10.3390/microorganisms13081881)
Supplement: Supplementary file 1 [file microorganisms-13-01881-s001.zip › microorganisms-3750231-supplementary.pdf]

Supplementary Materials

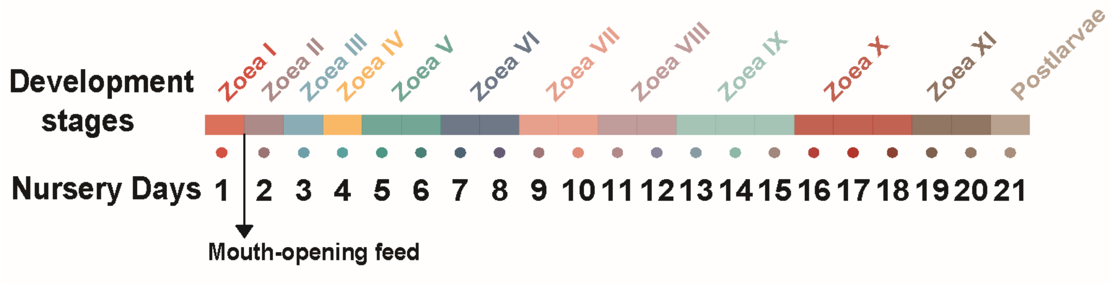

Figure S1. Experimental design and sampling schedules.

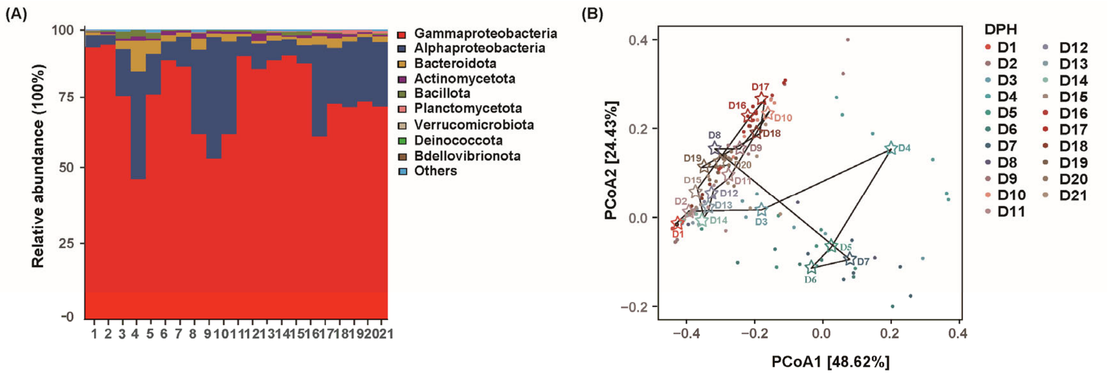

Figure S2. (**A**) Changes in the relative abundance of dominant phyla/proteobacterial classes (Top 10) of GFP larval bacterial communities. (**B**) Principal coordinate analysis (PCoA) visualizing compositional variations of larval bacterial communities across developmental stages based on Bray-Curtis dissimilarity.

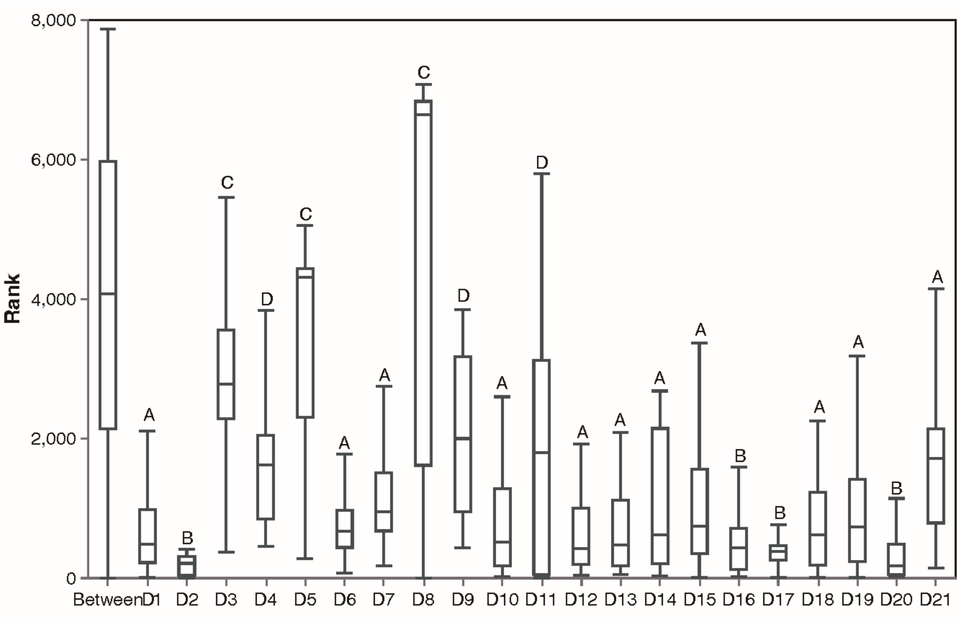

Figure S3. Analysis of microbial ANOSIM at each larval developmental stage of *Macrobrachium rosenbergii*. The different letters indicate a significant difference between stages ($p < 0.05$).
